# Controllable Surface Structures of Hydroxyapatite Processed by Picosecond Laser in Air and Underwater: A Comparative Study of Experiment and Simulation

**DOI:** 10.3390/ma19112379

**Published:** 2026-06-03

**Authors:** Li Liu, Peng Yao, Dongkai Chu, Shuoshuo Qu, Chuanzhen Huang

**Affiliations:** 1 Center for Advanced Jet Engineering Technologies (CaJET), School of Mechanical Engineering, Shandong University, Jinan 250061, China; 2 Key Laboratory of High Efficiency and Clean Mechanical Manufacture of the Ministry of Education, Jinan 250061, China; 3 School of Mechanical Engineering, Yanshan University, Qinhuangdao 066004, China

**Keywords:** hydroxyapatite, groove depth, groove width, electron temperature, lattice temperature, spatiotemporal evolution, comparison between experiment and simulation

## Abstract

Hydroxyapatite (HA) serves as an ideal in vitro substitute model for calcified plaques. At present, the influence mechanisms of processing parameters and operating environments on the machining morphology and thermal evolution of HA during picosecond laser processing remain unclear, and there is a lack of systematic analyses combining experiments and simulations. In this study, the effects of laser parameters and operating environments on structural parameters were systematically investigated from both experimental and simulation perspectives. The results demonstrate that within the laser energy range of 30–70 μJ, the groove depth and width are 12.1–47.8 μm and 15.6–32.1 μm in air, respectively, while they reach 15.4–48.6 μm and 22.4–47.3 μm underwater. Within the repetition frequency range of 100–140 kHz, the groove depth and width are 27.3–36.1 μm and 21.3–27.7 μm in air, respectively, compared with 34.6–45.4 μm and 33.3–53.3 μm underwater. The underwater-processed grooves exhibit larger dimensions and higher temperature-field values than those processed in air. Morphological observations further show that the groove bottoms formed in air exhibit bamboo-joint-like and granular features, whereas the underwater-processed grooves present a more uniformly distributed granular morphology. The simulation results agree well with the experimental data, with errors controlled within 12%, verifying the reliability of the established model. This study elucidates the morphological and thermal mechanisms of HA picosecond laser processing, supporting biomedical HA machining and paving the way for calcified plaque ablation and bone repair.

## 1. Introduction

HA is highly matched with severely calcified plaques in humans and natural bone tissue in terms of composition and mechanical properties. It is also a key raw material for the preparation of medical implants, making it an ideal substitute model for in vitro simulation studies [1,2,3,4]. Laser ablation has become a common technique for minimally invasive removal of calcified plaques, bone tissue repair, and precision machining of medical implants, and is widely used in the field of biological hard tissue processing [5,6,7,8,9]. However, calcified plaques are characterized by high hardness, high brittleness, and complex microstructures, and their safe and precise ablation remains a clinical challenge [10,11]. In addition, conventional laser processing tends to cause thermal damage, microcracks, and carbonized deposition in bone tissue and implants, with insufficient precision, which restricts its clinical application [12,13,14,15,16]. Ultrafast lasers represented by picosecond lasers feature ultra-short pulses and high peak power, offering a “cold processing” advantage in hard tissue machining and plaque ablation. They can suppress thermal diffusion and achieve low-damage precision ablation [17,18,19]. Therefore, a systematic study on the picosecond laser precision machining of HA as an in vitro surrogate model is of great theoretical value and clinical significance.

Most existing studies focus on the low-temperature characteristics of picosecond laser ablation and parameter regulation of surface structures. Jowett et al. confirmed through in vitro experiments that picosecond infrared lasers (PIRLs) induce a much lower surface temperature rise than traditional Er:YAG lasers during ablation of porcine skin and chicken humerus cortex while better preserving the tissue microstructure [20,21]. Körber et al. found that picosecond pulses can reduce the processing energy and thermal input by two orders of magnitude, and the shock wave pressure is also significantly lower than that of nanosecond pulses [22]. Zhan et al. demonstrated that increasing the scanning speed and optimizing the path can reduce the heat-affected zone and thermal damage [23]. Furthermore, tissue properties significantly affect the temperature rise effect: low-moisture hard tissues are prone to thermal accumulation under multi-pulse superposition [24], while water-cooling assistance can control the temperature rise during hard tissue ablation within a safe threshold, verifying the effectiveness of environmental regulation [25]. Although the low-temperature advantage of picosecond lasers has been verified, relevant studies are mostly concentrated in bone tissue engineering and mainly conducted in an air environment. Research on picosecond laser processing of HA is still insufficient, especially lacking the interpretation of the electron–lattice non-equilibrium thermal mechanism and systematic analysis combining experiments and simulations. Therefore, systematically exploring the intrinsic laws of picosecond laser ablation of HA and clarifying the regulation mechanisms of ambient media and process parameters can provide a solid theoretical basis and technical support for plaque ablation, bone tissue repair, and implant preparation.

In picosecond laser ablation simulations, the two-temperature model (TTM) is essential for describing the transient non-equilibrium energy transfer from the excited electronic subsystem to the lattice. Unlike the conventional single-temperature heat conduction model, the TTM can separately resolve electron and lattice temperature evolution during ultrashort laser–material interaction. Therefore, it provides a more physically appropriate framework for predicting temperature distribution, thermal accumulation, and ablation behavior in hydroxyapatite [26,27].

Based on the above research status and existing deficiencies, this paper systematically conducts studies on picosecond laser ablation of HA in air and underwater environments. It focuses on analyzing the spatiotemporal evolution characteristics of electron temperature and lattice temperature, the variation rules of ablation groove depth and width with laser fluence and repetition frequency, and verifies the reliability of the established model via comparative analysis of simulation data and experimental data. The results of this study can provide a solid theoretical basis and technical support for the optimization of minimally invasive, low-damage and high-precision laser ablation technology for severe atherosclerotic calcified plaques.

## 2. Materials and Methods

### 2.1. Picosecond Laser Processing System

**Figure 1:**
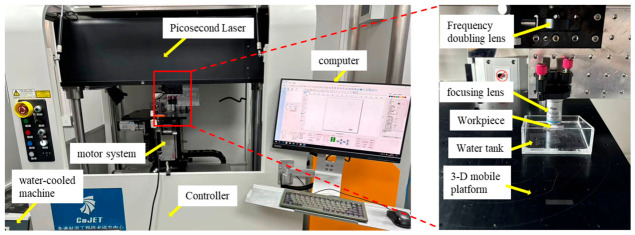
Photograph of the picosecond laser processing system.

The picosecond laser machining system used in this study was manufactured by GD Han’s Yueming Laser Group Co., Ltd. (Dongguan, China). The system mainly consisted of a picosecond laser source (BWT Tianjin Ltd. (Tianjin, China), BGL-1064-50B, wavelength 1064 nm, pulse duration 15 ps, adjustable repetition frequency range 10–1000 kHz, rated output power 50 W, and single-pulse energy > 500 μJ), a frequency-doubling module, and a four-axis precision motion platform. The frequency-doubling subsystem converts the laser wavelength from 1064 nm to 532 nm by integrating a frequency-doubled β-barium metaborate (BBO) crystal into the optical path. The photograph of the picosecond laser system employed in this study is illustrated in Figure 1.

### 2.2. Material and Experimental Scheme

In this study, HA disks (5 mm thick, 20 mm in diameter) were used as experimental materials. Comparative experiments in air and underwater were conducted to reveal the regulatory effect of the aqueous environment on the temperature distribution in the laser-affected zone [28,29]. The one-factor-at-a-time (OFAT) method was adopted to independently identify the effect of single parameters on temperature evolution. All the experiments were conducted in line-scanning mode at room temperature (25 ± 1 °C). The laser beam was focused onto the target surface using a 40× Olympus plan achromatic objective lens (PLN40X, NA = 0.65, focal length = 4.50 mm, working distance = 0.60 mm; Olympus Corporation, Tokyo, Japan). Before and after laser treatment, each sample was ultrasonically cleaned in anhydrous ethanol for 10 min to remove surface contaminants and debris. Three-dimensional surface topography was measured using a laser confocal microscope (LCM, VK-X200K, Keyence, Osaka, Japan). All dimensional data were averaged from at least ten measurement positions. The laser spot diameters on the target surface in air and underwater environments were determined by ablation threshold tests and were measured to be 19.38 μm and 24.84 μm, respectively. The single-pulse energies used in the experiments were 30, 40, 50, 60, and 70 μJ. The scanning speed was fixed at 1 mm/s for all the experiments. The detailed experimental parameters are listed in Table 1.

### 2.3. Numerical Simulation Setup

Numerical simulations were performed using the COMSOL Multiphysics 6.1 software. In this study, the classical electron–lattice two-temperature model (TTM) was employed as the core framework for numerical simulation, as presented in Equations (1) and (2). All the parameters used in the model were consistent with the experimental conditions. The actual laser processing parameters employed in the experiments, including laser wavelength, pulse duration, single-pulse energy, repetition frequency, laser spot diameter, and scanning speed, were introduced into the model. Meanwhile, key thermophysical parameters of HA, such as electron thermal conductivity, electron–lattice coupling coefficient, and electron heat capacity, were also incorporated. The underwater environment was simulated by setting the convective medium as deionized water to match the liquid environment in the experiments. The transient energy transfer between the electron and lattice subsystems was described using the two-temperature model, whose governing equations can be expressed as follows [30,31,32]:

(1) $$C_{e}(T_{e})\frac{\partial T_{e}}{\partial t}=▽\cdot {[k}_{e}(T_{e}){▽T}_{e}]-G\left(T_{e}-T_{l}\right)+S(r,t)$$

(2) $$C_{l}\frac{\partial T_{l}}{\partial t}=G\left(T_{e}-T_{l}\right)$$

where *T_e_* and *T_l_* are the electron and lattice temperatures, *C_e_* = *γT_e_* is the electron heat capacity, *C_l_* is the lattice heat capacity, *k_e_* is the electron thermal conductivity, *G* is the electron–phonon coupling factor, and *S*(*r, t*) represents the laser energy deposition source.

In the temperature field simulation of picosecond laser ablation of HA, the variation in scanning speed showed no obvious influence on the evolution of electron temperature and lattice temperature. Therefore, the variation trend with scanning speed was excluded in the simulation, and only laser fluence and repetition frequency were adopted as the key variables for analysis.

## 3. Results and Discussion

### 3.1. COMSOL Simulation Analysis of the Material Removal Mechanism

#### 3.1.1. Transient Temperature Evolution Based on the COMSOL Two-Temperature Model

**Figure 2:**
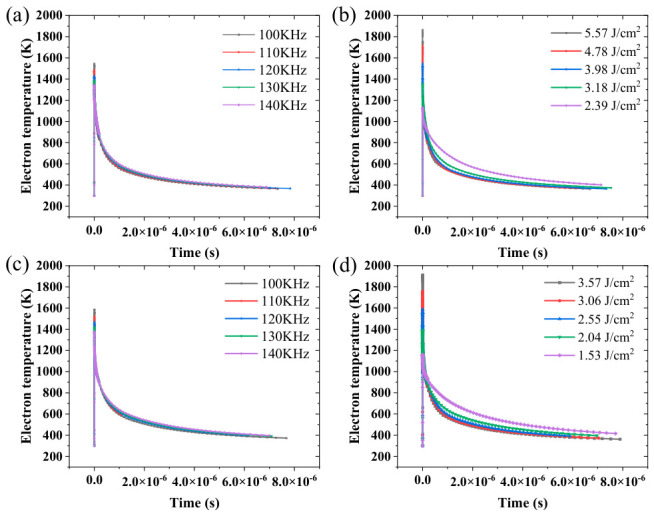
Electron temperature under different laser parameters in air and underwater: (**a**) electron temperature vs. repetition frequency in air, (**b**) electron temperature vs. laser fluence in air, (**c**) electron temperature vs. repetition frequency underwater, (**d**) electron temperature vs. laser fluence underwater.

**Figure 3:**
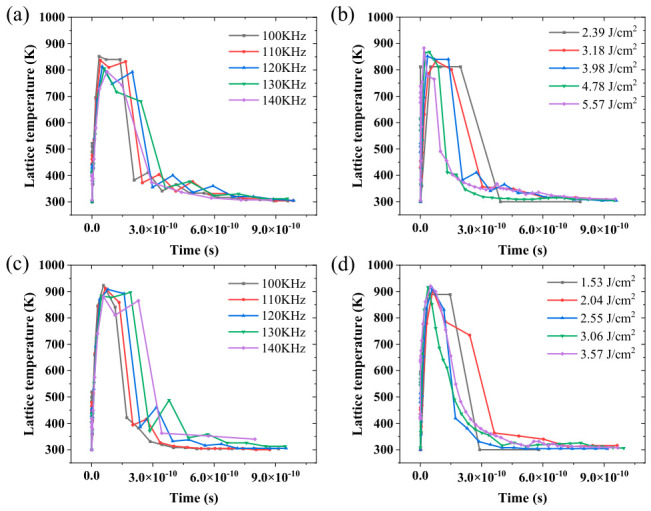
Lattice temperature under different laser parameters in air and underwater: (**a**) lattice temperature vs. repetition frequency in air, (**b**) lattice temperature vs. laser fluence in air, (**c**) lattice temperature vs. repetition frequency underwater, (**d**) lattice temperature vs. laser fluence underwater.

To clarify the ultrafast energy transfer process during picosecond laser ablation of hydroxyapatite, the double-temperature model was first employed to simulate the temporal evolution of electron and lattice temperatures. Figure 2 and Figure 3 respectively illustrate the evolution of electron temperature and lattice temperature in HA during picosecond laser ablation at different laser fluences and repetition frequencies in air and underwater. The electron temperature exhibits a typical three-stage evolution: it rises rapidly within 0–1 × 10^−12^ s, with the peak value ranging from 1340.8 to 1545.4 K in air and 1376.0–1585.9 K underwater with repetition frequency, and from 1129.1 to 1863.6 K in air and 1158.9–1912.5 K underwater with laser fluence. The peak increases with laser fluence but decreases with repetition frequency owing to the fixed total laser power and stronger localized energy accumulation under higher single-pulse energy, and the electron temperature underwater is notably higher than that in air. During the decay stage of 6 × 10^−11^–2 × 10^−9^ s, the temperature decreases continuously with an obvious inflection point that appears later under higher fluence or lower repetition frequency, and strong convective heat dissipation in water accelerates the temperature decay, especially under high initial energy conditions. After 1 × 10^−8^ s, the temperature differences among different groups gradually diminish, and all curves converge to similar values due to electron–lattice thermal equilibrium and enhanced heat dissipation in water, with the effects of fluence and repetition frequency being gradually weakened.

For the lattice temperature, it increases rapidly from 300 K in the initial ultrafast stage of 1 × 10^−12^–1 × 10^−11^ s, with the initial peak value ranging from 794.4 to 852.3 K in air and 881.2–923.4 K underwater with repetition frequency, and from 812.1 to 883.2 K in air and 888.7–920.8 K underwater with laser fluence. The peak shows the same dependence on fluence and repetition frequency as electron temperature and is also higher underwater than in air, which is dominated by the electron–phonon coupling process. At later times of 1 × 10^−11^–2 × 10^−10^ s, the lattice temperature fluctuates and decays obviously under high fluence but remains stable under low fluence, and the distinct heat dissipation modes further enlarge such differences: the weak conductive cooling in air versus the strong convective cooling underwater, while the local thermal confinement effect in water maintains a higher lattice temperature than in air under identical parameters. These results indicate that the rapid electron–lattice energy exchange governs the initial thermal response of HA and provides the physical basis for subsequent groove formation.

#### 3.1.2. Spatiotemporal Evolution of Electron and Lattice Temperatures During Groove Formation

**Figure 4:**
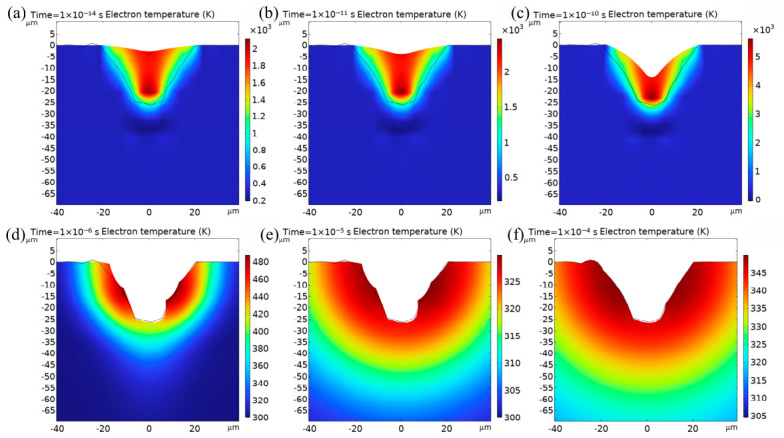
Spatiotemporal evolution of electron temperature in air: (**a**) 1 × 10^−14^ s, (**b**) 1 × 10^−11^ s, (**c**) 1 × 10^−10^ s, (**d**) 1 × 10^−6^ s, (**e**) 1 × 10^−5^ s, (**f**) 1 × 10^−4^ s.

**Figure 5:**
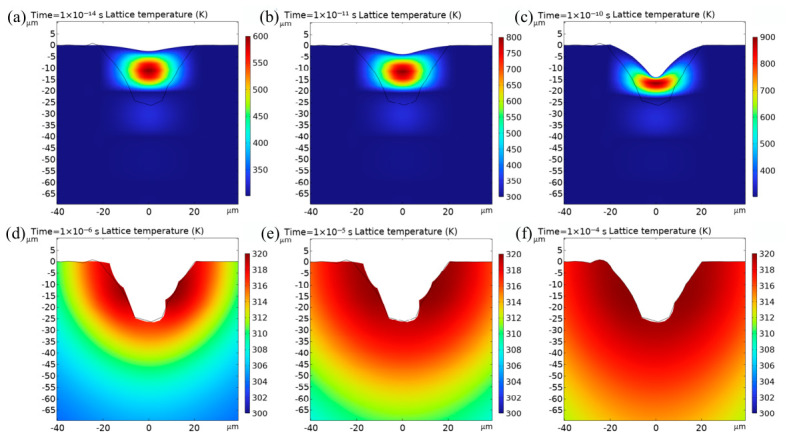
Spatiotemporal evolution of lattice temperature in air: (**a**) 1 × 10^−14^ s, (**b**) 1 × 10^−11^ s, (**c**) 1 × 10^−10^ s, (**d**) 1 × 10^−6^ s, (**e**) 1 × 10^−5^ s, (**f**) 1 × 10^−4^ s.

Based on the transient electron–lattice temperature evolution, the spatiotemporal temperature distribution during groove formation was further analyzed. The spatiotemporal evolution patterns of electron temperature and lattice temperature exhibit high similarity during picosecond laser ablation of HA in both air and underwater environments. Accordingly, this study focuses on the simulated spatiotemporal evolution results of electron temperature and lattice temperature in air, as presented in Figure 4 and Figure 5, respectively.

In this study, numerical simulations were performed to analyze the spatiotemporal evolution of electron temperature and the corresponding groove depth response during picosecond laser ablation of HA in air, with the results depicted in Figure 4. At the initial laser pulse stage, rapid localized energy deposition leads to a sharp increase in groove depth. During the decay stage, the electron temperature drops rapidly and diffuses into the surrounding medium, which reduces the material removal rate and slows down the growth of groove depth until it gradually saturates. In contrast to the evolution of groove depth, the groove width increases rapidly and approaches its maximum value at the initial stage. This is because the radial range of the laser spot is determined at the early stage of irradiation, and the lateral heat-affected zone and material removal region are directly limited by the spot size. Therefore, the groove width can reach saturation quickly, while the groove depth increases gradually with the continuous accumulation of pulse energy and the longitudinal penetration effect before finally stabilizing.

Both electron and lattice temperature peaks form at the subsurface rather than at the surface. This phenomenon is governed by three key factors: ultrahigh electron thermal conductivity, depth-dependent energy deposition, and electron–lattice coupling delay—the core feature distinguishing the TTM from conventional heat conduction models. Under ultrafast laser irradiation, electrons exhibit far higher thermal conductivity than the lattice system. Upon absorbing laser energy, electrons rapidly transfer energy inward via femtosecond-scale electron–electron scattering, creating a subsurface “thermal accumulation zone” instead of surface energy localization, which shifts the electron temperature peak inward over time. Laser energy is absorbed not only at the surface but also within a characteristic penetration depth, decaying exponentially with depth to form a volumetric heat source, thereby positioning the electron temperature peak at the subsurface location of maximum energy deposition. A second critical TTM feature is the strong electron–lattice non-equilibrium. Electron temperature rises sharply on the femtosecond timescale, while lattice temperature lags significantly due to its higher heat capacity and effective mass. Before lattice temperature increases substantially, electrons have already conducted energy inward, causing the lattice temperature peak to also occur at the subsurface. The simulated temperature fields indicate that material removal is initiated in the region with the highest energy deposition and gradually expands in both the depth and lateral directions. The evolution of the electron and lattice temperature distributions explains the formation process of the groove structure and clarifies how the groove depth and width are progressively established during laser ablation.

### 3.2. Simulation Analysis of the Influence Mechanism of Laser Parameters on Groove Characteristics

**Figure 6:**
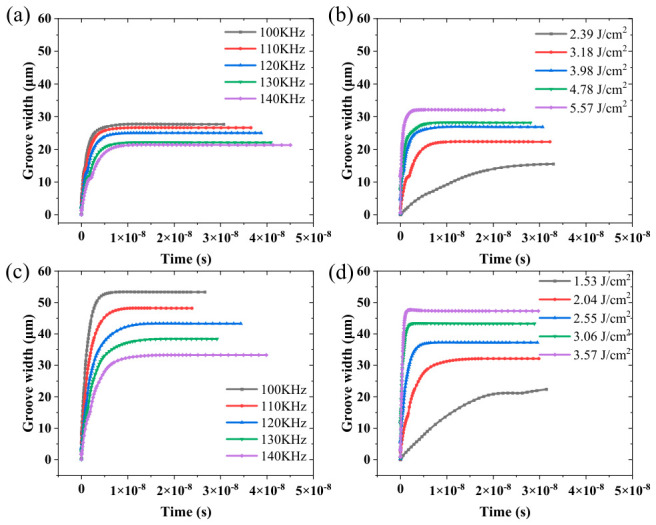
Variation trends of simulated groove width with laser fluence and repetition frequency in air and underwater: (**a**) groove width vs. repetition frequency in air, (**b**) groove width vs. laser fluence in air, (**c**) groove width vs. repetition frequency underwater, (**d**) groove width vs. laser fluence underwater.

**Figure 7:**
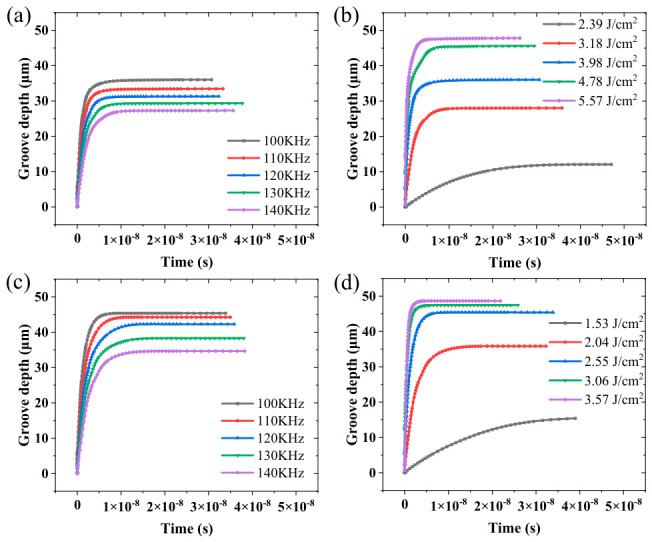
Variation trends of simulated groove depth with laser fluence and repetition frequency in air and underwater: (**a**) groove depth vs. repetition frequency in air, (**b**) groove depth vs. laser fluence in air, (**c**) groove depth vs. repetition frequency underwater, (**d**) groove depth vs. laser fluence underwater.

After clarifying the groove formation mechanism, the effects of laser parameters on groove morphology were quantitatively investigated through simulation. The simulated groove depth and width were extracted under different processing conditions to evaluate the regulatory effects of laser fluence, repetition frequency, and processing environment on groove characteristics. The evolution laws of groove width and depth during picosecond laser ablation of HA in both air and underwater environments are systematically revealed in Figure 6 and Figure 7, respectively. For groove dimensions, both width and depth present consistent evolutionary trends under varied process parameters in the two environments: they rise rapidly at the initial laser irradiation stage, and then the growth rate slows down gradually and tends to saturate eventually. For laser energies of 30–70 μJ, the groove width is 15.6–32.1 μm in air and 22.4–47.3 μm underwater, whereas the corresponding groove depth is 12.1–47.8 μm in air and 15.4–48.6 μm underwater. For repetition frequencies of 100–140 kHz, the groove width is 21.3–27.7 μm in air and 33.3–53.3 μm underwater, while the corresponding groove depth is 27.3–36.1 μm in air and 34.6–45.4 μm underwater. Specifically, laser fluence is positively correlated with both groove width and depth, while repetition frequency shows a negative correlation with them under constant total laser power, since higher energy density elevates single-pulse energy and strengthens material removal efficiency, whereas increased repetition frequency reduces single-pulse energy and weakens material removal capability. In addition, the plasma shielding effect and debris redeposition at high energy densities contribute to the final saturation of morphological dimensions [33,34,35,36]. Notably, groove depth is significantly more sensitive to the variation in energy density than groove width, with far larger growth amplitude and saturation value at high energy densities, verifying the more prominent penetration and accumulation effects of laser energy along the material depth direction. Underwater, laser fluence exhibits a more pronounced influence on groove dimensions due to stronger material removal and weaker thermal accumulation.

Compared with the air environment, the saturation values of both groove width and depth in the underwater environment are significantly higher, and the time to reach saturation is shorter. This is because the strong convective heat dissipation in water effectively suppresses material remelting and redeposition caused by heat accumulation. Meanwhile, the confinement effect of the aqueous medium enhances material removal by shockwaves and reduces plasma shielding, enabling more efficient delivery of laser energy to the material and thus improving the overall ablation efficiency [37,38]. Furthermore, the stronger heat dissipation in water avoids heat accumulation in the depth direction, allowing laser energy to be more effectively transferred into the material interior, which further strengthens the ablation effect in the depth direction [21,39,40,41,42].

The simulation results show that the groove depth and width vary systematically with laser parameters, indicating that the morphology of HA grooves can be effectively controlled by adjusting the processing conditions. These results further demonstrate that the proposed model can be used to predict the evolution of groove dimensions under different laser ablation environments.

### 3.3. Experimental Investigation of the Influence Mechanism of Laser Parameters on Structural Characteristics

**Figure 8:**
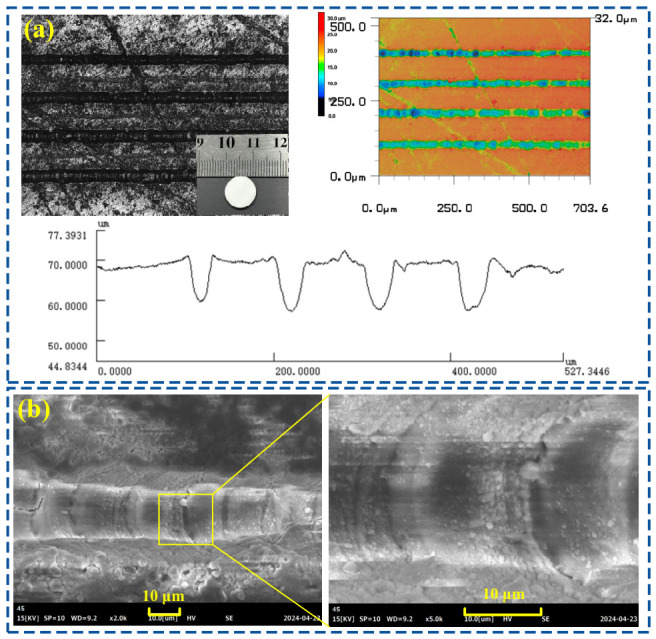
Representative experimental characterization of grooves produced by picosecond laser ablation of hydroxyapatite in air: (**a**) optical image of the processed sample, confocal microscopy image, three-dimensional surface morphology, and groove profile curve, and (**b**) SEM images of the laser-irradiated groove morphology.

**Figure 9:**
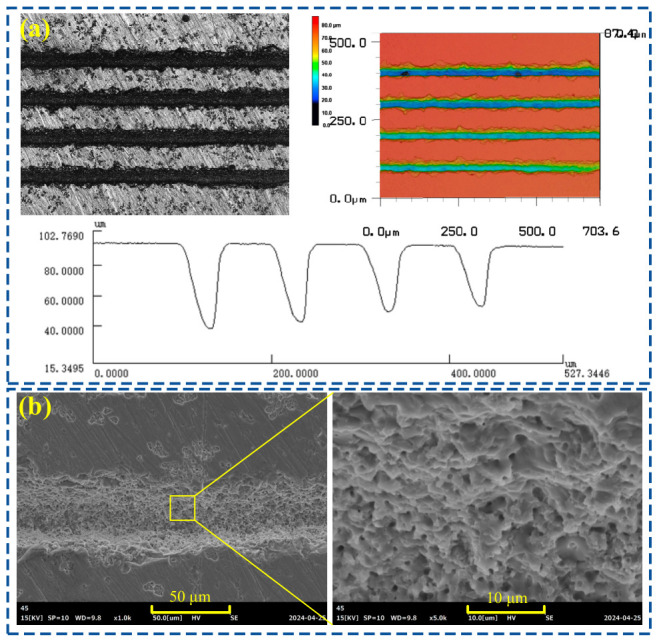
Representative experimental characterization of grooves produced by picosecond laser ablation of hydroxyapatite underwater: (**a**) confocal microscopy image, three-dimensional surface morphology, and groove profile curve, and (**b**) SEM images of the laser-irradiated groove morphology.

To verify the reliability of the simulation results, laser ablation experiments were conducted under corresponding processing conditions. The resulting groove morphology and elemental composition were characterized, and the experimentally measured groove dimensions were compared with the simulated results. Representative confocal microscopy and SEM images of the grooves fabricated in air and underwater are shown in Figure 8 and Figure 9. The confocal profiles were used to extract groove depth and width for quantitative analysis, while the SEM images reveal the surface morphology of the laser-irradiated regions. The groove bottom formed in air shows a bamboo-joint-like morphology with obvious granular and rough recast features, whereas the underwater-processed groove exhibits a more uniform granular structure. These results demonstrate the influence of the processing environment on groove formation and provide experimental evidence for comparison with the simulation results. This difference can be attributed to stronger heat accumulation and redeposition of molten ejecta in air, while rapid water cooling, plasma/plume confinement, and cavitation-assisted debris removal suppress recast accumulation and promote more homogeneous ablation underwater [43,44,45,46].

**Figure 10:**
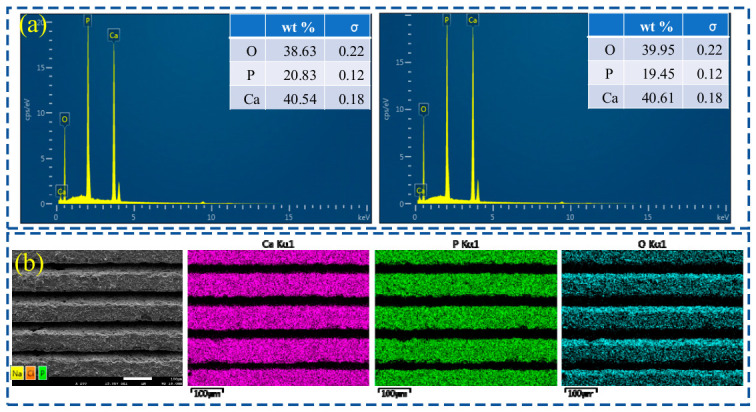
Elemental analysis of laser-processed HA surfaces in air and underwater: (**a**) EDS spectra and elemental contents of the grooves processed in air and underwater and (**b**) SEM image and corresponding elemental mapping images of Ca, P, and O in the laser-processed grooves.

The elemental composition of the laser-processed HA surfaces in air and underwater was analyzed by EDS, as shown in Figure 10. The EDS spectra indicate that the main elements of HA, including O, P, and Ca, are retained after laser processing under both environments. The elemental contents obtained in air and underwater show only slight differences, suggesting that the processing environment has no obvious influence on the overall elemental composition of the HA surface. In addition, the elemental mapping results further confirm that Ca, P, and O are uniformly distributed in the laser-processed grooves.

**Figure 11:**
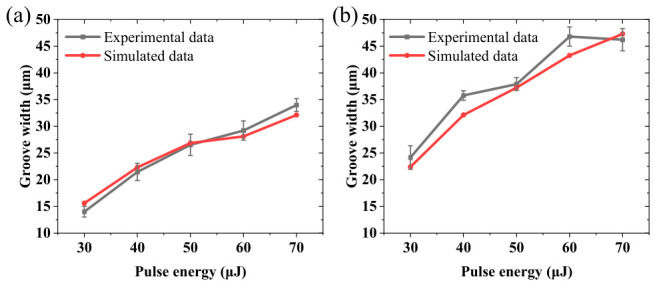
Comparison of simulated and experimental results for groove width under different pulse energies: (**a**) in air and (**b**) underwater.

**Figure 12:**
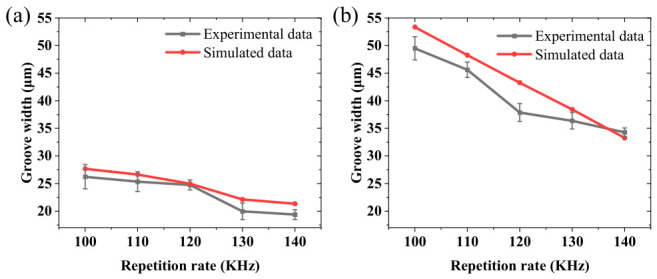
Comparison of simulated and experimental results for groove width under different repetition frequencies: (**a**) in air and (**b**) underwater.

**Figure 13:**
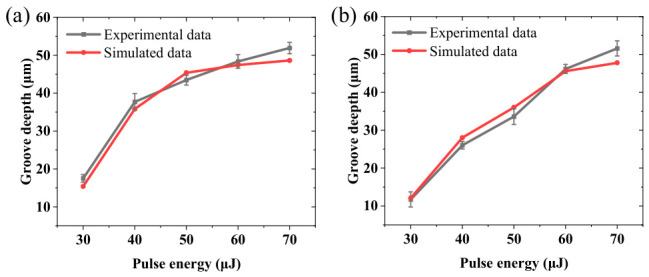
Comparison of simulated and experimental results for groove depth under different pulse energies: (**a**) in air and (**b**) underwater.

**Figure 14:**
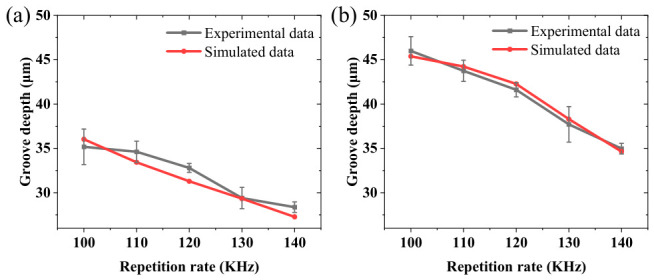
Comparison of simulated and experimental results for groove depth under different repetition frequencies: (**a**) in air and (**b**) underwater.

Figure 11, Figure 12, Figure 13 and Figure 14 compare the simulated and experimental groove width and depth during picosecond laser ablation of HA in air and underwater, respectively. The corresponding simulated and experimental data for groove width and depth are provided in detail in the Supplementary Materials. In both air and underwater environments, the groove width increased with increasing laser fluence and showed an overall decreasing trend with increasing repetition frequency. This trend can be mainly attributed to the reduction in single-pulse energy and peak intensity at higher repetition frequencies under the present experimental conditions, which weakened the lateral ablation of hydroxyapatite. The groove depth rises rapidly with laser fluence before gradually plateauing, and declines monotonically with repetition frequency. Overall, the simulated and experimental results exhibit highly consistent trends and close values in all cases. The maximum relative differences are 6.67% at 30 μJ (width, air), 11.11% at 130 kHz (width, air), 5.88% at 70 μJ (depth, air), 3.55% at 140 kHz (depth, air), 12.00% at 30 μJ (width, underwater), 6.67% at 100 kHz (width, underwater), 5.26% at 30 μJ (depth, underwater), and 2.27% at 130 kHz (depth, underwater). Overall, the simulated groove width and depth agree well with the experimental results, with the maximum difference ratios controlled within 12% and 6% respectively, which verifies the reliability and accuracy of the established numerical model in predicting the evolution laws of groove geometry during laser processing.

The relative deviations between the simulation and experiment in both the air and underwater environments are controlled within 12%, with the agreement for groove depth being generally better than that for groove width. Compared to the air environment, the maximum deviation in groove width slightly increases underwater, which is mainly attributed to the more complex effects of strong convective heat dissipation, liquid-phase flow, and debris scouring in water, leading to certain randomness in the thermal influence and material removal behavior at the edge region. In contrast, the groove depth is less affected by environmental disturbances, and its simulation–experiment deviation is close to that in air, further confirming that the model provides a more accurate description of energy penetration and material removal in the depth direction. Overall, the surface morphology, elemental analysis, and simulation–experiment comparison collectively verify the formation mechanism of laser-ablated grooves on HA and demonstrate the reliability of the proposed model for predicting material removal behavior in both air and underwater environments.

## 4. Conclusions

This study systematically investigates the spatiotemporal evolution of temperature and the parametric dependence of ablation morphology during picosecond laser ablation of HA in air and underwater environments, and validates the reliability of the simulation model. The results reveal that in both environments, the electron temperature rises instantaneously and decays rapidly to room temperature under pulsed irradiation, while the lattice temperature evolves in stages. Strong convective heat dissipation in water accelerates temperature decay and mitigates heat accumulation. Regarding ablation morphology, both groove depth and width increase rapidly and then saturate with increasing laser fluence, and show an overall decreasing trend with rising repetition rate. Groove depth is more sensitive to laser fluence, highlighting the penetration and accumulation of laser energy along the depth direction. Underwater ablation, supported by the synergistic effects of weakened remelting–redeposition and enhanced shockwave removal, achieves larger saturated dimensions and shorter saturation times. Surface morphology observations further show that the grooves formed in air mainly exhibit a bamboo-joint-like morphology, whereas underwater ablation produces a more uniform particle-distribution morphology. In addition, elemental analysis indicates that the elemental contents after ablation in air and underwater environments change only slightly, suggesting that the processing environment has limited influence on the overall elemental composition of HA. Comparisons between the simulation and experimental data show that the maximum errors in groove depth and width are below 12%, with better fitting consistency for groove depth, confirming the high accuracy and strong applicability of the two-temperature and material removal models in describing laser etching under different environments. Overall, the numerical and experimental results provide a basis for understanding the temperature evolution and groove formation mechanisms during picosecond laser ablation of hydroxyapatite in air and underwater environments. Future studies will focus on coupling the simulation model with comprehensive post-processing material characterization and biological evaluations, thereby further guiding parameter optimization and promoting its potential applications in biomedical material processing.

## Figures and Tables

**Table 1:** Linear ablation experimental parameters.

Order Number	Scanning Speed /(mm/s)	Energy /(μJ)	Laser Fluence in Air /(J/cm^2^)	Laser Fluence Underwater /(J/cm^2^)	Repetition Rate /KHz
1	1	30	2.39	1.53	100
2		40	3.18	2.04	110
3		50	3.98	2.55	120
4		60	4.78	3.06	130
5		70	5.57	3.57	140

## Data Availability

The original contributions presented in this study are included in the article/Supplementary Materials. Further inquiries can be directed to the corresponding authors.

## Supplementary Materials

The following supporting information can be downloaded at: https://www.mdpi.com/article/10.3390/ma19112379/s1, Figures S1: Confocal microscopy-derived groove profiles of hydroxyapatite after picosecond laser ablation under different processing conditions; Figures S2: Confocal microscopy images of laser-processed grooves on hydroxyapatite; Figures S3: Representative measurement of groove depth and width using VK analysis software based on confocal microscopy data. The non-English terms shown in the VK software interface refer to the measurement/analysis items displayed by the software; Figures S4: Simulated groove width and groove depth evolution in air under different processing conditions; Figures S5: Simulated groove width and groove depth evolution underwater under different processing conditions. Table S1: Summary of experimental and simulated groove width and groove depth data under different laser fluence and repetition frequency conditions in air and underwater environments.
