# Supplementary material for: Controllable Surface Structures of Hydroxyapatite Processed by Picosecond Laser in Air and Underwater: A Comparative Study of Experiment and Simulation
Source: Materials (Basel). 2026 Jun 3;19(11):2379. doi: 10.3390/ma19112379 (PMC13258162; doi:10.3390/ma19112379)
Supplement: Supplementary file 1 [file materials-19-02379-s001.zip › materials-4351653-supplementary.pdf]

Supplementary Measurement Images for Figures 11-14

Due to the addition and reorganization of figures in the revised manuscript, the original Figures 8 and 9 have been renumbered as Figures 11-14. This supplementary file provides the original confocal microscopy measurement images used to obtain the groove depth and groove width data presented in Figures 11-14.

These images include representative measurements of grooves produced in both air and underwater environments under different laser fluence and repetition frequency conditions. The groove profiles extracted from these confocal images were used for quantitative comparison between the experimental results and the simulation results. In addition, the summarized experimental and simulated data used for the comparison in Figures 11-14 are provided in Table S1. Figures S1–S3 present the experimental measurement-related images, including the confocal microscopy morphology images, groove profile curves, and representative VK analysis software measurement interface. Figures S4 and S5 present the simulation-related results corresponding to the simulated data shown in the main manuscript. These supplementary figures and the data summary table provide additional support for the comparison between the experimental measurements and simulation results.

Table S1. Summary of experimental and simulated groove width and groove depth data under different laser fluence and repetition frequency conditions in air and underwater environments.

Category	Air–Repetition Frequency	Air–Laser Fluence	Underwater–Repetition Frequency	Underwater–Laser Fluence	Air–Repetition Frequency	Air–Laser Fluence	Underwater–Repetition Frequency	Underwater–Laser Fluence
	Groove Width (μm)				Groove Depth (μm)			
Experimental Data	26.23	14.01	49.49	24.15	35.18	11.73	46	17.51
	25.34	21.46	45.61	35.77	34.62	26.04	43.75	37.7
	24.74	26.53	37.86	37.9	32.8	33.6	41.61	43.45
	19.97	29.21	36.37	46.8	29.4	46.18	37.71	48.39
	19.38	33.98	34.28	46.21	28.37	51.59	34.97	51.92
Simulated Data	27.68	15.6	53.34	22.41	36.03	12.09	45.38	15.4
	26.62	22.3	48.26	32.12	33.43	28.02	44.23	35.83
	24.98	26.88	43.27	37.24	31.29	36.03	42.27	45.38
	22.12	28.1	38.42	43.26	29.33	45.59	38.3	47.42
	21.34	32.1	33.27	47.3	27.28	47.8	34.64	48.62

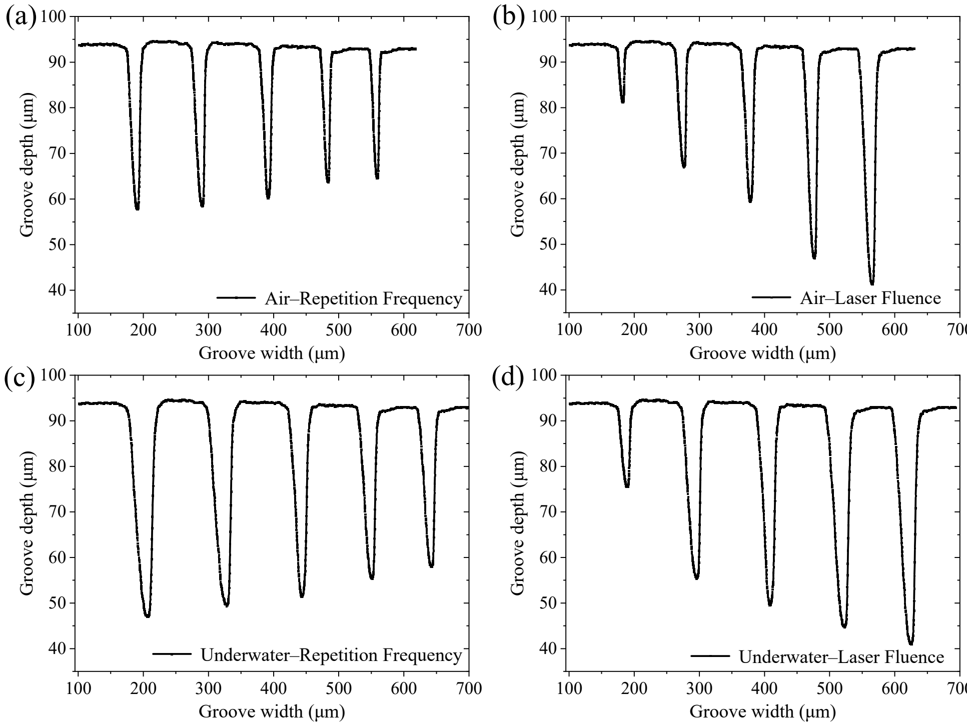

Figure S1. Confocal microscopy-derived groove profiles of hydroxyapatite after picosecond laser ablation under different processing conditions: (a) air–repetition frequency; (b) air–laser fluence; (c) underwater–repetition frequency; (d) underwater–laser fluence.

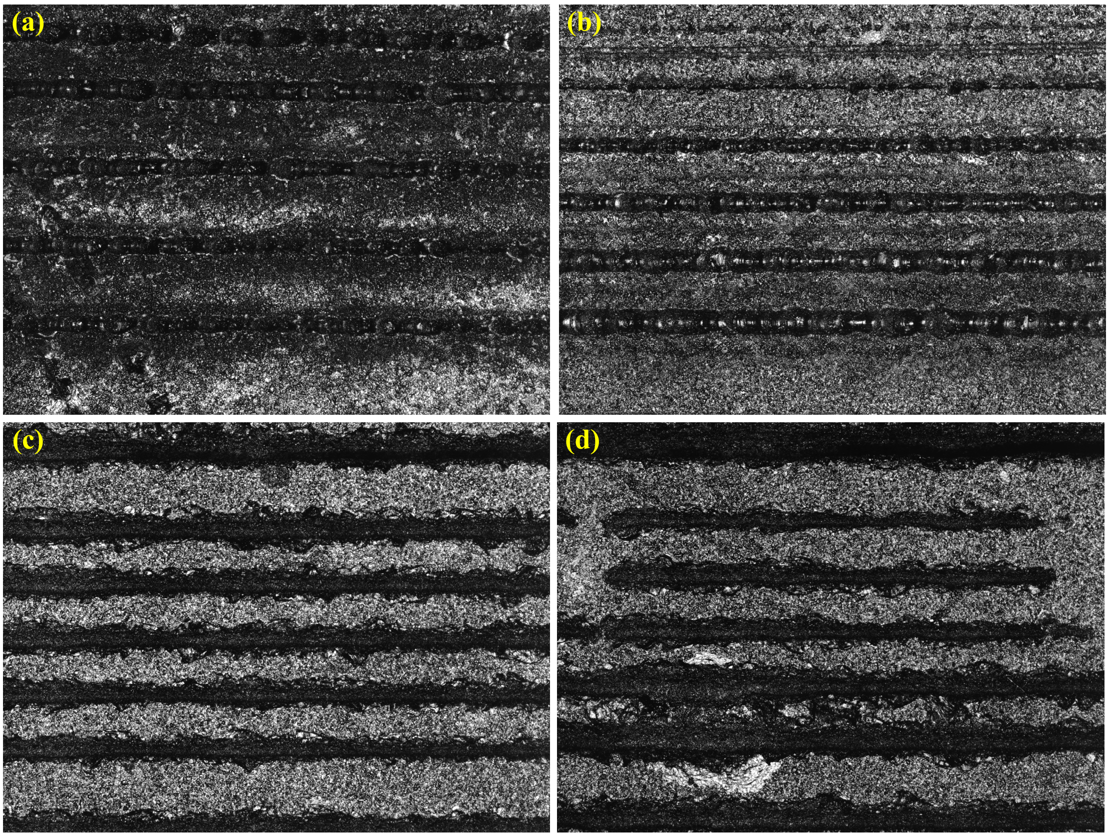

Figure S2. Confocal microscopy images of laser-processed grooves on hydroxyapatite: (a) air–repetition frequency; (b) air–laser fluence; (c) underwater–repetition frequency; (d) underwater–laser fluence.

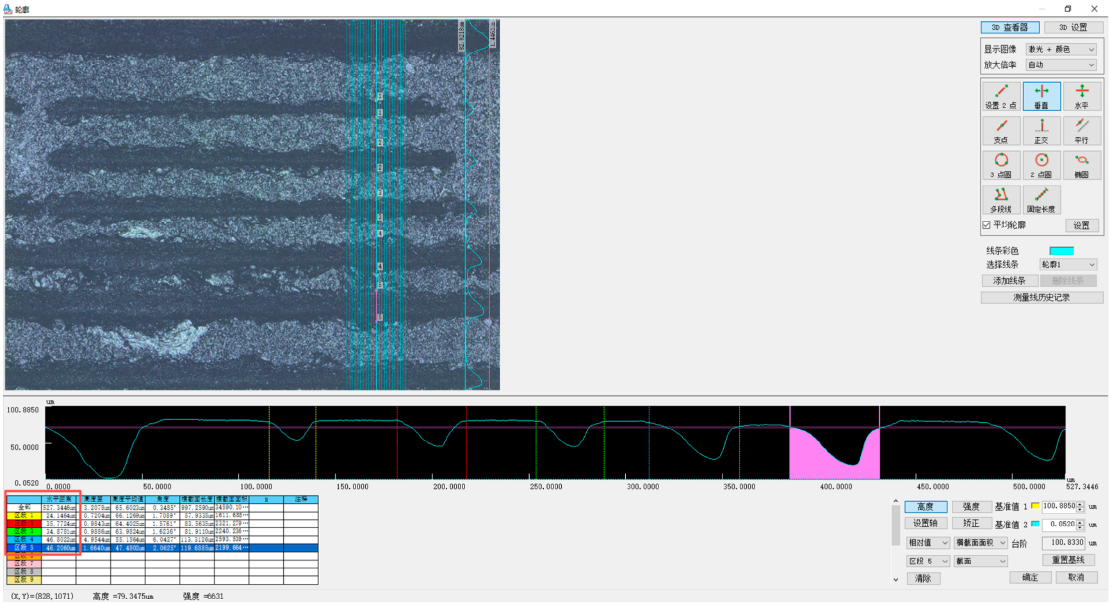

Figure S3. Representative measurement of groove depth and width using VK analysis software based on confocal microscopy data. The non-English terms shown in the VK software interface refer to the measurement/analysis items displayed by the software.

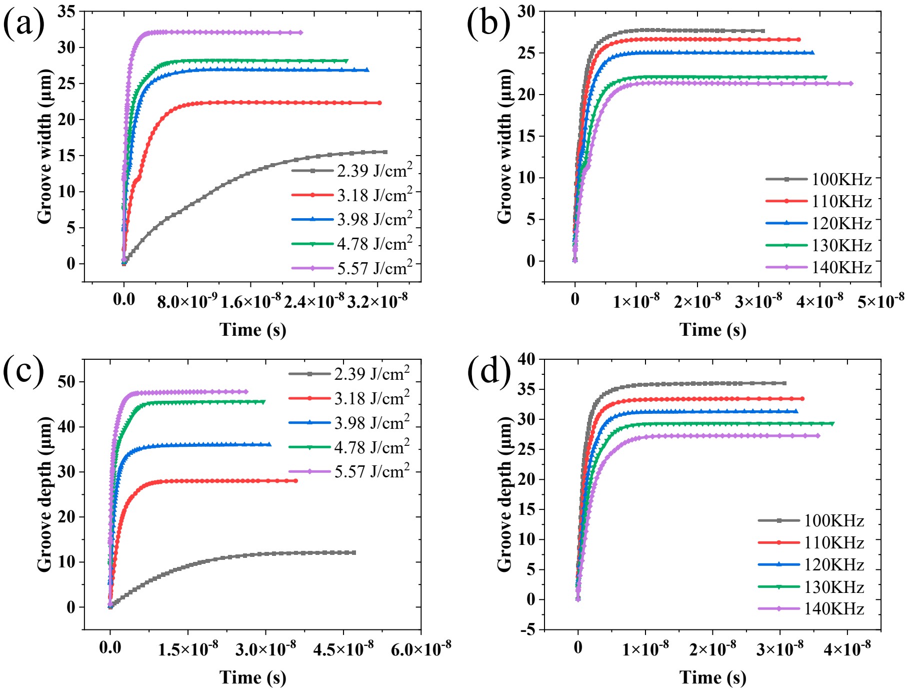

Figure S4. Simulated groove width and groove depth evolution in air under different processing conditions: (a) groove width–laser fluence; (b) groove width–repetition frequency; (c) groove depth–laser fluence; (d) groove depth–repetition frequency.

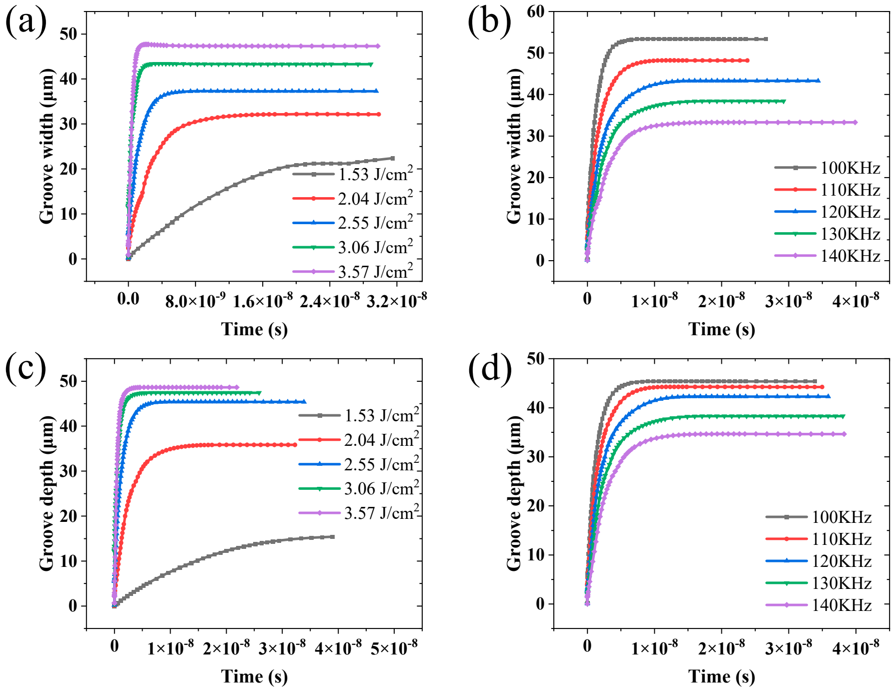

Figure S5. Simulated groove width and groove depth evolution underwater under different processing conditions: (a) groove width–laser fluence; (b) groove width–repetition frequency; (c) groove depth–laser fluence; (d) groove depth–repetition frequency.
